# MR Angiography Follow-Up 10 Years after Cryptogenic Nonperimesencephalic Subarachnoid Hemorrhage

**DOI:** 10.1371/journal.pone.0117925

**Published:** 2015-02-17

**Authors:** Holger Wenz, Gregory Ehrlich, Ralf Wenz, Mohamad-Motaz al Mahdi, Johann Scharf, Christoph Groden, Peter Schmiedek, Marcel Seiz-Rosenhagen

**Affiliations:** 1 University Medical Center Mannheim, University of Heidelberg, Department of Neuroradiology, Mannheim, Germany; 2 University Medical Center Mannheim, University of Heidelberg, Department of Neurosurgery, Mannheim, Germany; St Michael's Hospital, University of Toronto, CANADA

## Abstract

**Objectives:**

Long-term magnetic resonance angiography (MRA) follow-up studies regarding cryptogenic nonperimesencephalic subarachnoid hemorrhage (nSAH) are scarce. This single-centre study identified all patients with angiographically verified cryptogenic nSAH from 1998 to 2007: The two main objectives were to prospectively assess the incidence of *de novo* aneurysm with 3.0-MRI years after cryptogenic nSAH in patients without evidence for further hemorrhage, and retrospectively assess patient demographics and outcome.

**Methods:**

From prospectively maintained report databases all patients with angiographically verified cryptogenic nSAH were identified. 21 of 29 patients received high-resolution 3T-MRI including time-of-flight and contrast-enhanced angiography, 10.2 ± 2.8 years after cryptogenic nSAH. MRA follow-up imaging was compared with initial digital subtraction angiography (DSA) and CT/MRA. Post-hemorrhage images were related to current MRI with reference to persistent lesions resulting from delayed cerebral ischemia (DCI) and post-hemorrhagic siderosis. Patient-based objectives were retrospectively abstracted from clinical databases.

**Results:**

29 patients were identified with cryptogenic nSAH, 17 (59%) were male. Mean age at time of hemorrhage was 52.9 ± 14.4 years (range 4 – 74 years). 21 persons were available for long-term follow-up. In these, there were 213.5 person years of MRI-follow-up. No *de novo* aneurysm was detected. Mean modified Rankin Scale (mRS) during discharge was 1.28. Post-hemorrhage radiographic vasospasm was found in three patients (10.3%); DCI-related lesions occurred in one patient (3.4%). Five patients (17.2%) needed temporary external ventricular drainage; long-term CSF shunt dependency was necessary only in one patient (3.4%). Initial DSA retrospectively showed a 2 x 2 mm aneurysm of the right distal ICA in one patient, which remained stable. Post-hemorrhage siderosis was detected 8.1 years after the initial bleeding in one patient (4.8%).

**Conclusion:**

Patients with cryptogenic nSAH have favourable outcomes and do not exhibit higher risks for *de novo* aneurysms. Therefore the need for long-term follow up after cryptogenic nSAH is questionable.

## Introduction

In approximately 15% (range 5–30%) of all patients suffering from spontaneous subarachnoid hemorrhage (SAH) no source of bleeding can be detected despite initial four vessel cerebral digital subtraction angiography (DSA) [1,2]. This subset of patients can be divided into two subgroups according to the bleeding pattern on initial cranial CT. Two-thirds show a perimesencephalic pattern, involving the midbrain cisterns with no evidence of intraventricular or intracerebral bleeding. This group is associated with a benign course and excellent short-term and long-term prognosis as well as very low incidence of rebleeding [1,3,4]. Recent evidence suggests a venous cause for most of these hemorrhages [5]. On the other hand, the nonperimesencephalic SAH (nSAH) mimics a pattern that is common in aneurysmal subarachnoid hemorrhage. It affects the Sylvian fissure, carotid cisterns, and/or interhemispheric fissure. This entity is characterized by absence of abnormalities in DSA. According to Wermer *et al*. [6], 2.3% of patients with SAH caused by a ruptured aneurysm develop a *de novo* aneurysm. Furthermore, enlargement of conservatively treated aneurysms have been reported to occur in 25% (mean interval of 8.9 years) [6]. However, little is known about the frequency of *de novo* aneurysm formation after cryptogenic nSAH. In a recent study by Pyysalo *et al*. [2] 33 patients were examined with 1.5 T-MRI more than 9 years (mean 12 years) after SAH of unknown aetiology. Although there was no distinction between the nonperimesencephalic and perimesencephalic type of SAH, no *de novo* aneurysms were detected in MRA images within this cohort [2].

The policy in our department has changed after a single case of cryptogenic nSAH that presented 7 years later with a ruptured aneurysm of the anterior communicating artery [7]. Since then a routine MRA scan after 5 years is implemented in the follow-up of these patients. But to the best of our knowledge there are no long-term studies analysing intracranial arterial status after cryptogenic nSAH. Therefore, this reported case poses the question: do *de novo* aneurysms occur years after cryptogenic nSAH? In order to elucidate this question, we prospectively assessed the incidence of *de novo* aneurysm formation after cryptogenic nSAH with 3 Tesla MRI in a long term follow-up in event-free patients with former cryptogenic nSAH. The secondary objective was to retrospectively investigate related general patient demographics, complication rates and discharge conditions of these patients.

## Methods

### Patients demographics and study design

The study was approved by the local institutional review board (IRB, Medizinische Ethikkommission II der Medizinischen Fakultät Mannheim der Universität Heidelberg, Mannheim, Germany). This study consisted of a retrospective and a prospective part. For the retrospective part of our study, patient consent was not required according to our IRB for the de-identified database (Syngo Data Manager—SDM) and due to the lack of patient interaction. For the prospective part of our study, patients were included after obtaining their written informed consent.

For the inclusion into the study we identified all patients with angiographically verified former cryptogenic nSAH and event-free further course from 1998 to 2007 constituting a pool of about 520 SAH patients (which were non-traumatic and non-perimesencephalic). Exclusion criteria were trauma, blood coagulation disorders or drug abuse (for example cocaine). 29 patients met all criteria.

Retrospectively, we examined all 29 patients and evaluated the following parameters: (1) clinical outcome, (2) mortality, (3) need for temporary external ventricular drainage, (4) long-term shunt dependency, (5) mean hospital stay, (6) mean intensive care unit (ICU) stay, (7) radiographic vasospasm, and (8) delayed cerebral ischemia (DCI). These data were abstracted from a clinical database.

Within the prospective component of the study, 21 of the total 29 patients were examined, as the remaining 8 could not participate in further MRI investigations due to either missing documents, death (not related to initial SAH or re-bleeding), refusal to participate or MRI contraindications. Prospectively, we examined the following four parameters: (1) mean follow-up years, (2) person years of MRI-follow-up, (3) siderosis in MRI and (4) *de novo* aneurysm.

### Magnetic resonance imaging

Imaging was performed on a 3T scanner (Magnetom Trio; Siemens Healthcare, Erlangen, Germany). A standard 12 channel head and neck coil was used and all images were acquired with subject’s head and neck in a neutral position. The imaging protocol consisted of a TOF-MRA (**T**ime-**o**f-**F**light **M**agnetic **R**esonance **A**ngiography; slice thickness of 0.5 mm, TR 20 msec, TE 3,59 msec), a ceMRA (**c**ontrast-**e**nhanced **M**agnetic **R**esonance **A**ngiography; slice thickness of 1 mm, TR 3,10 msec, TE 1,21 msec), an axial T2-TIRM (T2-weighted **T**urbo-**I**nversion **R**ecovery-**M**agnitude) sequence, an axial T2-TSE (T2-weighted double echo **T**urbo **S**pin **E**cho image), an axial T1-TIRM df (T1- weighted **T**urbo-**I**nversion **R**ecovery-**M**agnitude **d**ark **f**luid image), an axial DWI (**D**iffusion-**W**eighted-**I**mage) and an axial T2*-weighted image. Contrast enhanced images were acquired using a body weight adapted dose of gadoteric acid (Dotarem, Guerbet, Aulnay-sous-Bois, France).

### Image evaluation

All viewing and analysis of the acquired datasets were performed electronically on a DICOM workstation using multidimensional image navigation and display software OsiriX (OsiriX Imaging Software; Advanced Open-Source PACS Workstation DICOM viewer, http://www.osirix-viewer.com/index.html). The software was used for TOF-MRA/ceMRA to reconstruct 2D multiplanar reconstruction images in axial, coronal, and sagittal planes [8]. Two experienced neuroradiologists (H.W., J.S.) analysed each dataset in consensus, searching for intracranial aneurysmatic lesions in TOF-MRA and ceMRA. Axial T2* images were used for detection of posthemorrhagic siderosis. MRA follow-up imaging was compared with DSA, MRA or CT angiography at the time of hemorrhage. Furthermore both investigators reviewed all follow-up investigations immediately after bleeding (DSA, CT, MRI) recording vasospastic abnormalities in DSA and post-hemorrhage lesions resulting from DCI in CT and current MRI. Clinical standard operating procedure searching for an aneurysm consists of a 4-vessel DSA including internal carotid artery (ICA) and vertebral artery on both sides. In case of negative finding, it is common practice to investigate also the external carotid arteries in order to exclude dural arteriovenous fistulas as bleeding source.

### Statistical analysis

Patient characteristics and clinical outcome parameters were given as n (%), mean values and standard deviation (±). These were analyzed using Microsoft Office Excel 2010 (Microsoft Corporation 2010, Redmond, USA; http://www.microsoft.com). In order to calculate the relative incidence of *de novo* aneurysm in our patient cohort of moderate size, we employed the exact method for calculating the 95% confidence interval. The confidence interval for this paper was generated using SAS software version 9.3 (SAS Institute Inc., Cary, NC).

## Results

### Demographics and initial diagnosis

**Fig 1:**
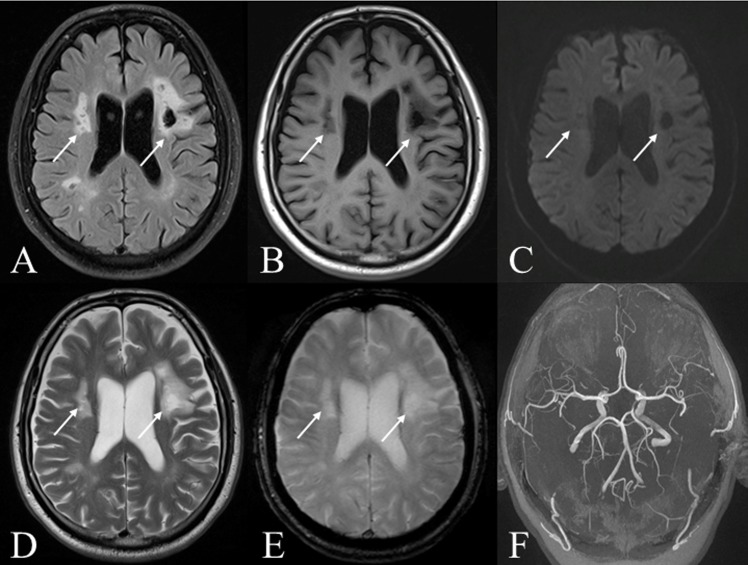
MRI follow-up six years after nonperimesencephalic subarachnoid hemorrhage of a 59-year-old male patient (Table 1, #15). DCI-related lesions are indicated by arrows. *A-E* show bilateral chronic liquor-isointense areas with surrounding gliosis and no diffusion restriction, nor hemosiderin residues. *F* depicts no aneurysm in TOF-angiography. *Sequences*: *A =* T2 TIRM weighted, *B* = T1-TIRM weighted, *C* = diffusion-weighted, *D* = T2 weighted, *E* = T2* weighted, *F* = time-of-flight-angiography.

We identified 29 patients with a single event of a cryptogenic nSAH, 17 (59%) patients were male, 12 were female (41%). In the whole database there was only one—already published [7]—patient with a former cryptogenic nSAH that presented again with a ruptured aneurysm of the anterior communicating artery. Mean age at the time of hemorrhage was 52.9 ± 14.4 years (range 4 to 74 years). During the initial admittance to hospital, 11 patients (37.9%) out of 29 patients received CTA in addition to initial CT scan, 2 patients (6.9%) underwent subsequent MRA. All patients received a DSA during period of hospitalization. Repeated DSA was performed in 7 patients (24%), repeated CTA in one patient (5%). In 3 out of 29 patients radiographic vasospasm was detected (10.3%), either in the initial DSA or in a control after bleeding. DCI-related lesions occurred in one patient (3.4%) (Fig. 1). There was a need for a temporary external ventricular drainage in 5 (17.2%) patients. Long-term CSF shunt dependency was necessary only in one patient (3.4%). Mean modified Rankin Scale (mRS) on discharge was 1.28 (±0.53). Mean hospital stay was 10.7 days (±7.17); mean intensive care unit (ICU) stay was 2.3 days (±3.22).

**Table 1:** Clinical details of patients with cryptogenic nonperimesencephalic subarachnoid hemorrhage.

Patient	Sex	Age at bleeding	Year of bleeding	Year of follow-up MRI	tEVD	lSD	rVS	DCI	mRS	mHS	mICUS	FU yrs	AA	Siderosis
1	2	73	1999	°	1	1	0	0	2	29	2	°	°	°
2	1	68	2001	°	1	0	0	0	2	16	7	°	°	°
3	1	55	2005	2014	1	0	1	0	1	17	0	9,33	0	0
4	1	62	2001	°	0	0	0	0	3	13	0	°	°	°
5	2	60	2000	°	1	0	0	0	1	28	11	°	°	°
6	1	52	2001	°	0	0	0	0	2	8	6	°	°	°
7	1	47	2007	2013	0	0	0	0	1	8	4	6,83	0	0
8	2	42	2003	2013	0	0	0	0	1	6	0	10,17	0	0
9	2	66	2001	2013	0	0	0	0	2	0	2	11,75	0	0
10	2	52	1997	2013	0	0	0	0	1	3	0	16,92	0	0
11	2	61	2002	2013	0	0	0	0	1	6	0	11,33	0	0
12	2	54	2007	2013	0	0	0	0	1	6	0	6,25	0	0
13	1	67	2003	2013	0	0	0	0	1	10	0	10,50	0	0
14	2	41	2003	2013	0	0	0	0	1	5	0	10,42	0	0
15	1	53	2007	2013	0	0	1	1	2	5	2	6,17	0	0
16	1	47	1998	2013	0	0	0	0	1	7	2	14,67	0	0
17	1	65	2005	2013	0	0	0	0	1	2	0	8,08	0	1
18	2	41	2000	2013	0	0	0	0	1	3	0	12,67	0	0
19	1	58	1999	2013	0	0	0	0	1	9	0	14,33	0	0
20	1	58	2006	2013	0	0	0	0	2	0	2	6,58	0	0
21	2	36	2005	2013	0	0	0	0	1	3	2	8,17	1	0
22	1	51	2003	2013	1	0	0	0	1	5	7	10,17	0	0
23	1	74	2003	2013	0	0	0	0	1	9	0	10,50	0	0
24	1	56	2006	2013	0	0	0	0	1	4	2	7,75	0	0
25	1	58	2004	2013	0	0	1	0	1	3	11	8,83	0	0
26	2	4	2001	2013	0	0	0	0	1	3	0	12,08	0	0
27	2	64	2004	°	0	0	0	0	1	15	2	°	°	°
28	1	35	2005	°	0	0	0	0	1	14	4	°	°	°
29	1	34	2005	°	0	0	0	0	1	8	0	°	°	°

1 = male, 2 = female, tEVD = need of an temporary external ventricular drainage, lSD = long term shunt dependency, rVS = radiographic vasospasm, DCI = delayed cerebral ischemia, mRS = mean Rankin scale, mHS = mean hospital stay *(day)*, mICUS = mean intensive care unit stay (day), FU yrs = follow-up years, AA = aneurysm,° = follow-up data missing

### Follow-up demographics and MRI-analysis

**Fig 2:**
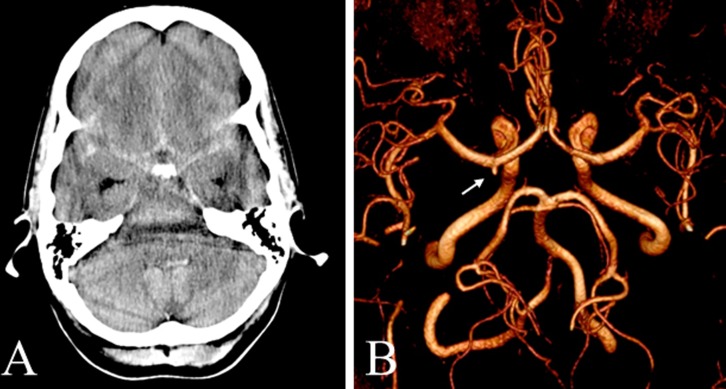
Initially undetected aneurysm found 8 years after SAH of a 44-year-old female patient (Table 1, #21). *A* CT scan at admission in 2005 revealing nSAH in the frontal interhemispheric fissure, in the premedullary cisterns and the bilateral sylvian fissures. The consecutive digital subtraction angiography was false negative. *B* Intracranial TOF-angiogram of the same patient in July 2013 demonstrating an aneurysm of the right internal carotid artery *(arrow)* which was retrospectively visible in the initial post-hemorrhagic digital subtraction angiography (Fig. 3).

**Fig 3:**
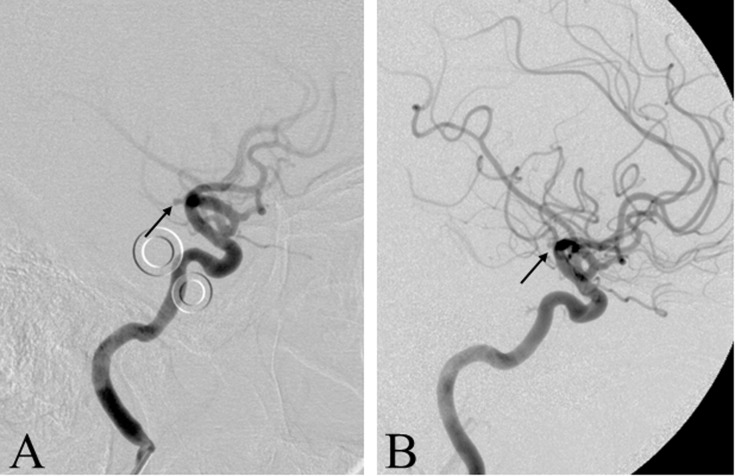
Initial and follow-up digital subtraction angiography of a 44-year-old female patient (Table 1, #21) 8 years after bleeding. *A* Right ICA angiogram (lateral view) obtained in 2005 depicting a 2 x 2 mm aneurysm of the right distal ICA *(arrow)*, which was not initially detected. *B* Pre-operative angiogram of the right ICA (lateral view) in 2013; since 2005 the morphology and extension of the distal ICA aneurysm remained stable *(arrow)*. ICA = internal carotid artery.

In 21 patients, high-resolution 3T-MRI was performed 10.2 ± 2.8 years after cryptogenic nSAH. There were 213.5 person years of follow-up. 12 (57%) patients were male, 9 were female (43%). Mean age at the time of hemorrhage was 51.7 ± 14.6 years. In one patient, follow-up MRA 8 years after bleeding showed a 2 x 2 mm aneurysm of the right distal ICA (Fig. 2). This aneurysm was retrospectively visible in the initial post-hemorrhage DSA. Since then its morphology and extension were stable (Fig. 3). In this patient no further imaging was performed after initial angiography. As the patient suffered from SAH, the aneurysm was successfully treated by clipping.

**Fig 4:**
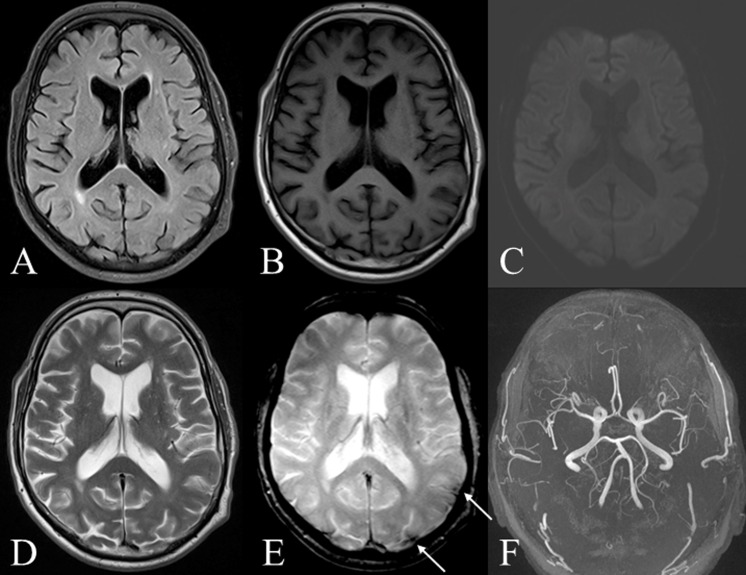
MRI follow-up eight years after nonperimesencephalic subarachnoid hemorrhage of a 73-year-old male patient (Table 1, #17). Post-hemorrhagic sulcal siderosis is indicated by arrows. *A-D* show no residuals after bleeding. *E* shows sulcal siderosis on the parietal and occipital left hemisphere. *F* depicts no aneurysm in TOF-angiography. Sequences: *A* = T2 TIRM weighted, *B* = T1-TIRM weighted, *C* = diffusion-weighted, *D* = T2 weighted, *E* = T2* weighted, *F* = time-of-flight-angiography.

Susceptibility-weighted T2*- imaging 8.1 years after bleeding revealed post-hemorrhage siderosis in one patient (4.8%) (Fig. 4). Within our prospective study population, no *de novo* aneurysm was detected. Calculating the 95% confidence interval of *de novo* aneurysm rate showed a lower confidence limit of 0% and an upper confidence limit of 16.11%.

## Discussion

In approximately 15% of all patients suffering from SAH, no bleeding source can be detected using initial DSA [1,2]. Moreover, little is known about the *de novo* aneurysm frequency after cryptogenic nSAH. In the present study, there was a low yield in detecting *de novo* aneurysm using MRA 10 years after nSAH.

Although our study comprises of a limited number of patients, to our knowledge it is the first study with a prospective study design to focus on the incidence of *de novo* aneurysm after nSAH. Our multimodal 3T-MRA technique with and without intravenous contrast enhancement provides high-resolution images of the intracranial vessels [9–12]. The absence of artefacts due to coils or clips makes it unlikely that aneurysms were missed [11]. Although reports on the incidence of *de novo* aneurysm formation after cryptogenic nSAH are scarce, our study is mostly in concordance with recently published data on incidence of *de novo* aneurysms after aneurysmal SAH: A valid database is represented by the International Subarachnoid Aneurysm Trial (ISAT), which provides a follow up database of 2143 patients with ruptured intracranial aneurysms [13]. Within this study, rebleeding from a *de novo* aneurysm was reported in six cases with a subsequent incidence of about 0.28% [13]. Similarly, Lindvall *et al*. (2012) [14], Plowman *et al*. (2011) [15], So *et al*. (2010) [16] and Sprengers *et al*. (2009) [17] report *de novo* aneurysm rates of 0%, 0.2%, 0% and 1.5% (mean follow-up years of 9.7, 3.13 and 1.8).

Within our prospective cohort, no patient (0%) developed a *de novo* aneurysm or other vascular pathology in the long term follow-up. Nonetheless, the calculation of the confidence interval shows that the *de novo* aneurysm rate is likely to be between 0% and 16.11%, which can be assumed with a probability of 95%. While we could find no *de novo* aneurysm, we could find a previously undetected small aneurysm of the right distal ICA in one patient, which was retrospectively already visible in post-hemorrhage DSA. This aneurysm was then successfully treated by clipping. In general, DSA has a sensitivity of 99% and a negative predictive value of 96% to 98% according to the literature [18–20]. Therefore, the one patient in our study with an initially “false negative” DSA would have benefitted from a second DSA after bleeding. Within our cohort, only 7 patients underwent a second DSA. It was common practice in our hospital to obtain initial CTA after detection of a SAH followed by a DSA. In some cases a second DSA was performed to rule out post-hemorrhagic vasospasm. However, in light of the current study, second DSA work-up after initial DSA appears to be beneficial at least for the assessment of post-hemorrhagic vasospasm. Accordingly, further work-up after initial negative DSA in 47 patients with nSAH revealed vascular pathologies in 16%, as shown by Andaluz *et al*. [1]. Similarly, a diagnostic yield of 10.0% could be achieved after initial negative baseline assessment in a pooled analysis of repeated DSA in 368 patients [21]. These initially false negative results further emphasize the importance of a prompt and aggressive diagnostic work-up that might lead to a second or even a third angiogram [1,7,22,23]. The timeframe of further work-up and the respective yield of additional DSA were investigated by Dalyai *et al*. [24]. The authors investigated the efficacy of short (1-week)—and long-term (6-week) repeated angiography in 136 cases of negative initial catheter angiography in nSAH. They found aneurysms in repeated DSA in a high percentage (12.5%) [24]. Interestingly, the yield of short- and long-term repeated angiographic examinations was nearly similar [24].

The secondary objective was to retrospectively investigate complication rates and mean hospital stay of the patients. Comparing the complications rates and demographics in nSAH and aneurysmal SAH we could add weight to the conclusion of previous investigators [24,25]: In our cohort, radiographic vasospasm was found in three patients (10.3%); hemodynamic relevant vasospasm leading to DCI occurred in one patient (3.4%). Similar to our results, a recent study of Dalyai *et al*. reports of an incidence of angiographic vasospasm in 13.2% within 254 SAH cases with negative initial DSA [24]. Additionally, according to Fontanella *et al*., nSAH shows transcranial Doppler acceleration suspicious for vasospasm in 11.1%, clinical relevant vasospasm in 3.2% and no DCI (0%) [25]. In contrast, the incidence of increased Doppler velocity after aneurysmal SAH is 27%, clinical vasospasm 18% and ischemia 7.1% respectively [25]; which indicates that aneurysmal SAH has a higher rate of associated complications than nSAH.

Moreover, symptomatic hydrocephalus is another complication following non-aneurysmatic SAH: a 0 to 15% incidence of hydrocephalus requiring surgical intervention has been reported for patients with angiogram-negative SAH [26]. Although transient increases in ventricular size are very common, the need for a definitive CSF-shunt internalization is rare [26–28]. In our cohort, five patients (17.2%) were treated with a temporary external ventricular CSF drainage suffering from symptomatic hydrocephalus; long-term dependency and ventriculo-peritoneal shunting was found only in one patient (3.4%).

Furthermore, we investigated the mean hospital stay of the patients and compared these to the literature. In our study, mean hospital stay was 10.7 days, mean ICU stay 2.2 days. This is similar to a US cohort of Andaluz *et al*. reporting a mean hospital stay of 8.3 and mean ICU stay of 5.4 days [1]. In contrast, the Italian study of Fontanella *et al*. reports a mean hospitalization of 22.4 days [25]. Differences between our study and Andaluz’ study on the one hand and Fontanella’s study on the other hand might reflect differences in medical health care systems or internal hospital policies. Nonetheless, we acknowledge that for some of our patients there are very short hospitalization periods. However, this might reflect a general trend to transfer patients early into special neuro-rehabilitation centers if a vascular pathology is excluded and the patient is in a good clinical status.

The present study has some limitations. First, our study sample is of moderate size. Even though this size is larger than any other available studies on the subject, it is not large enough for drawing definite conclusions regarding the utility of late screening in this nSAH patient collective. Second, due to the considerable time difference between initial diagnosis and recent follow-up, some data records are incomplete or inhomogeneous (e.g. hard copies of the event instead of computer based images). Third, as it is in most single centre studies, there might be a selection bias, sample bias, or image-based selection bias [29]. Lastly, some studies postulated discrepancies in detecting aneurysms in DSA and MRA. However, in these studies MRI scans did not follow a standard protocol and were obtained in various different facilities [30]. In contrast, we used one protocol on a 3 Tesla MRI, which has been repeatedly shown to be highly reliable [9–12]. Thus, an additional invasive imaging procedure was judged inappropriate with the availability of a non-invasive reliable diagnostic method. Nonetheless, it cannot be excluded that especially a very small aneurysm was overlooked in the MRI follow up, which would have been detected with DSA.

In conclusion, our results are in concordance with previous literature, which states that nSAH has a more benign course than aneurysmal SAH in terms of complications. According to our investigation, a long-term follow-up MRI is not showing any *de novo* aneurysms or other potential bleeding sources in nSAH patients. Thus its routine use remains questionable in the first instance. However, the calculated probability of the *de novo* aneurysm formation after nSAH ranges from 0% to 16.11%. Therefore, there is need for studies with larger population sizes further assessing the incidence of *de novo* aneurysms and the optimal timing of further diagnostic in patients with nSAH is warranted. Nonetheless, prompt aggressive work-up including repeated early DSA remains strongly recommended in the initial diagnostic procedure.
